# Contrast timing optimization of a two-volume dynamic CT pulmonary perfusion technique

**DOI:** 10.1038/s41598-022-12016-8

**Published:** 2022-05-17

**Authors:** Yixiao Zhao, Logan Hubbard, Shant Malkasian, Pablo Abbona, Sabee Molloi

**Affiliations:** Department of Radiological Sciences, Medical Sciences I, B-140, University of California, Irvine, Irvine, CA 92697 USA

**Keywords:** Preclinical research, Lung cancer, Medical research, Experimental models of disease

## Abstract

The purpose of this study is to develop and validate an optimal timing protocol for a low-radiation-dose CT pulmonary perfusion technique using only two volume scans. A total of 24 swine (48.5 ± 14.3 kg) underwent contrast-enhanced dynamic CT. Multiple contrast injections were made under different pulmonary perfusion conditions, resulting in a total of 141 complete pulmonary arterial input functions (AIFs). Using all the AIF curves, an optimal contrast timing protocol was developed for a first-pass, two-volume dynamic CT perfusion technique (one at the base and the other at the peak of AIF curve). A subset of swine was used to validate the prospective two-volume pulmonary perfusion technique. The prospective two-volume perfusion measurements were quantitatively compared to the previously validated retrospective perfusion measurements with t-test, linear regression, and Bland–Altman analysis. As a result, the pulmonary artery time-to-peak (${T}_{PA}$) was related to one-half of the contrast injection duration ($\frac{{T}_{Inj}}{2}$) by ${T}_{PA}=1.01\frac{{T}_{Inj}}{2}+1.01$ (r = 0.95). The prospective two-volume perfusion measurements (P_PRO_) were related to the retrospective measurements (P_RETRO_) by P_PRO_ = 0.87P_RETRO_ + 0.56 (r = 0.88). The CT dose index and size-specific dose estimate of the two-volume CT technique were estimated to be 28.4 and 47.0 mGy, respectively. The optimal timing protocol can enable an accurate, low-radiation-dose two-volume dynamic CT perfusion technique.

## Introduction

Computed tomography (CT) has enabled the non-invasive quantification of pulmonary perfusion allowing for the assessment of pulmonary embolism and pulmonary hypertension^1–5^. Existing dynamic CT perfusion techniques require the entire contrast pass curve over many cardiac cycles for perfusion measurement, resulting in high radiation dose^2,3,6–8^. Moreover, the pulmonary perfusion measured by such techniques is known to be underestimated due to the use of small tissue volumes for measurement^9,10^. Although dual-energy CT iodine map is also used to depict pulmonary perfusion defects, it has limited contrast-to-noise ratio and cannot provide absolute pulmonary blood flow measurement ^11–14^. Hence, an accurate, low-dose dynamic CT perfusion technique is necessary for improved physiological assessment of pulmonary disease.

Fortunately, previous studies have demonstrated that accurate cardiac and pulmonary perfusion measurement is feasible with a first-pass analysis (FPA) technique using only two volume scans^9,10,15^: one at the base (V1) and one at the peak (V2) of the arterial input function (AIF). Nevertheless, these prior validations required the entire AIF curve and retrospectively down-sampled to two volume scans for blood flow measurement. Hence, a timing protocol for the true prospective implementation of two-volume FPA technique remains necessary, where such protocol can also account for different hemodynamic conditions and cardiac outputs^16,17^.

Thus, the purpose of this study is to develop an optimal timing protocol for the prospective two-volume FPA dynamic CT pulmonary perfusion technique. The central hypothesis is that the time interval between the two volume scans can be pre-determined using the contrast injection parameters and an empirical time constant. Finally, using the proposed timing protocol, the accuracy of the two-volume prospective FPA dynamic CT perfusion technique was assessed and compared to the previously validated retrospective FPA perfusion technique^15^.

## Methods

### Ethical statement and ARRIVE guideline

**Figure 1:**
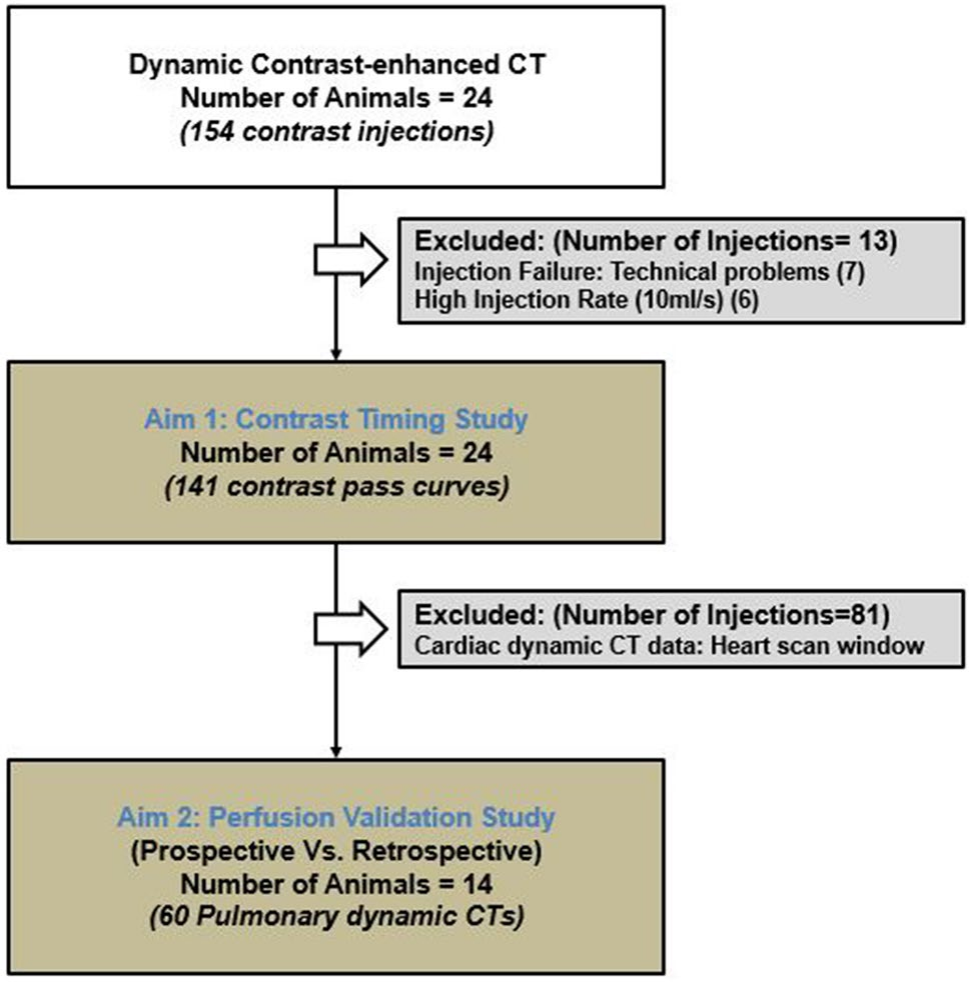
Flowchart of the study. A total of 141 successful contrast injections were used in the Aim1 study in investigate the optimal acquisition time of the maximal contrast enhancement in pulmonary artery. A total of 60 pulmonary CT acquisitions were used in the prospective two-volume perfusion validation study.

All experiments were performed in accordance with the ARRIVE guidelines. The study was approved by the Institutional Animal Care and Use Committee (IACUC, Protocol Number: AUP-18-191) at University of Irvine, California. A total of 24 male Yorkshire swine (48.5 ± 14.3 kg) were used with 154 contrast injections, where thirteen were excluded due to injection failures and high injection rates. For aim 1, a total of 141 successful contrast injections were used to retrospectively develop an optimal timing protocol for the two-volume pulmonary perfusion technique. Specifically, the time-to-peak delay between the baseline and the peak time of the pulmonary arterial enhancement curve was predicted based on the contrast injection duration and a dispersion time constant. For aim 2, prospective two-volume pulmonary perfusion technique was simulated in a subset of fourteen swine with 60 contrast injections using the predicted time-to-peak, where the remaining 81 injections were excluded as they were originally acquired as part of a cardiac perfusion study with heart scan window (Fig. 1). The accuracy of the two-volume prospective technique was compared to the previously validated retrospective perfusion measurement^15^. All experimental data was prospectively acquired by all authors between March 2016 and December 2017 and was retrospectively analyzed between June 2018 and July 2019. Y.Z., L.H. and Sh.M. conducted the data analysis, and a radiologist with more than 15 years of clinical experience (P.A.) conducted the surgical and interventional procedures. The data that support the findings of this study are available from the corresponding author, SM, upon reasonable request. S.M., has previously received grants from Canon America Medical Systems. The remaining authors have nothing to disclose.

### Animal preparation

All 24 swine were premedicated with Telazol (4.4 mg/kg), Ketamine (2.2 mg/kg) and Xylazine (2.2 mg/kg) then intubated (Mallinckrodt, tube 6.0–8.0 mm, Covidien, Mansfield, MA). Anesthesia was maintained with 1.5–2.5% Isoflurane (Baxter, Deerfield, IL) in oxygen via mechanical ventilation (Surgivet, Norwell, MA, and Highland Medical Equipment, Temecula, CA). Two femoral venous and one femoral arterial introducer sheaths (5-Fr AVANTIR, Cordis Corporation, Miami Lakes, FL) were placed for intravenous contrast medium injection, fluid administration, and arterial pressure monitoring, respectively. An introducer sheath and Swan-Ganz catheter were then placed into a distal pulmonary arterial branch, via the jugular vein, under fluoroscopic guidance for the eventual induction of balloon occlusion. The cardiac output was varied by producing balloon occlusion in the left caudal lobe at different locations of the pulmonary artery. On average six pulmonary perfusion studies were performed during each experiment. At the conclusion of each experiment, all animals were euthanized with saturated KCI.

### CT imaging protocol

Contrast material (Isovue 370, Bracco Diagnostics, Princeton, NJ) was injected followed by a saline chaser (Empower CTA, Acist Medical Systems, Eden Prairie, MN). Multiple contrast injections were made for each animal and different injection rates and volumes were used as shown in Table 1. ECG-gated dynamic scanning was then performed with a 320-slice CT scanner (Aquilion One, Canon America Medical Systems, Tustin, CA) for approximately 30 cardiac cycles during a ventilator-controlled inspiratory breath hold. The following scan parameters were used: tube voltage, 100 kVp; tube current, 200 mA; detector collimation, 320 × 0.5 mm; volume scanning mode; gantry rotation time, 0.35 s; slice thickness, 0.5 mm; scan field-of-view, 240–400 mm; voxel raster, 512 $\times$ 512; and a FC07 soft tissue reconstruction kernel with AIDR3D iterative reconstruction. A 20-minute time delay was used between all acquisitions to allow for adequate contrast material recirculation and redistribution.

**Table 1:** Contrast injection protocols.

Injection protocols	Iodine dose per body weight (ml/kg)	Saline chaser (ml/kg)	Injection rate (ml/s)
Protocol A	1	0.5	5
Protocol B	0.5	0.25	5

### Aim 1—time-to-peak delay estimation

**Figure 2:**
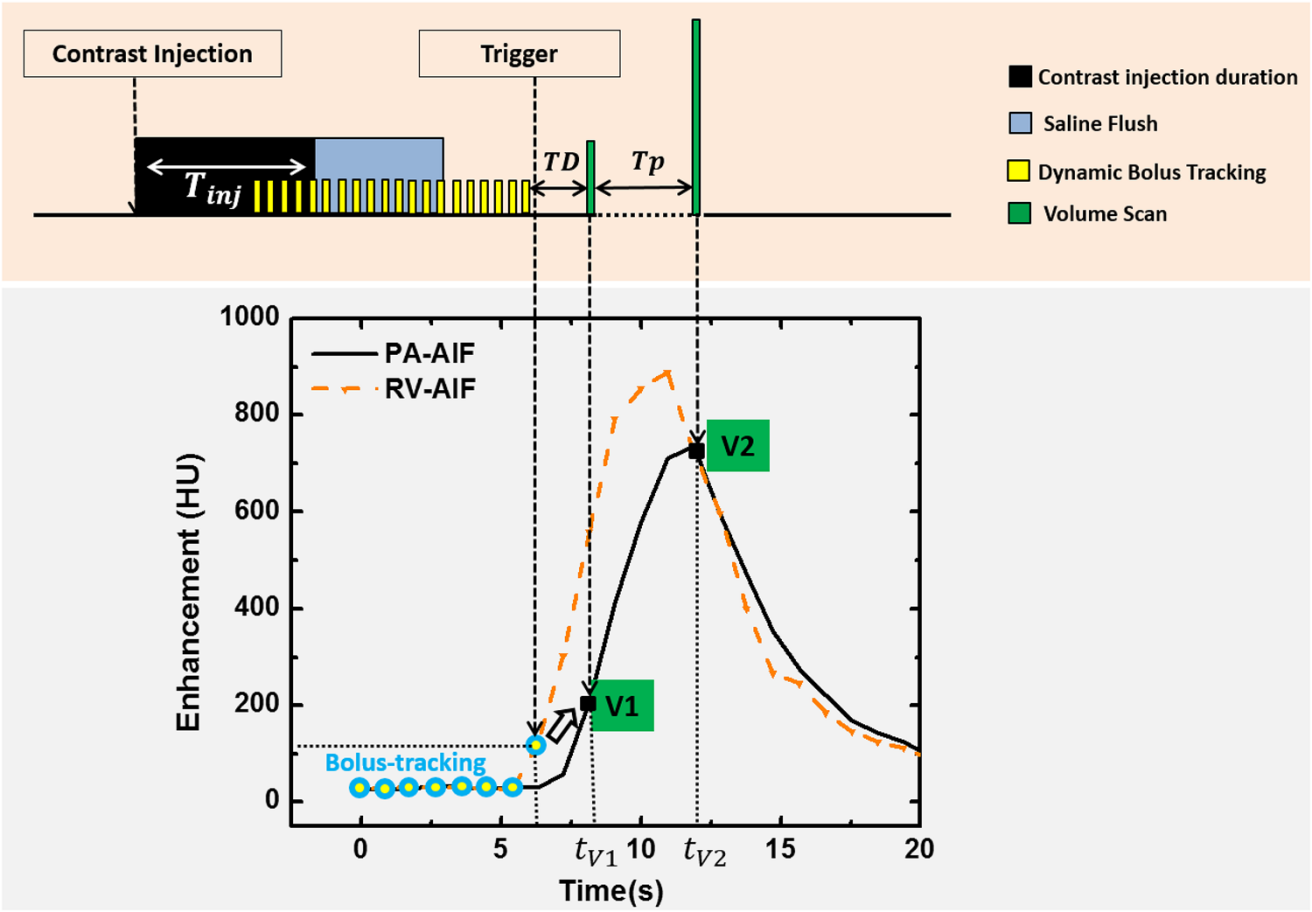
Prospective imaging protocol and the corresponding arterial input functions. Top, imaging protocol. ${\mathbf{T}}_{\mathbf{i}\mathbf{n}\mathbf{j}}$ is the contrast injection duration, $\mathbf{T}\mathbf{D}$ is the scanner-specific transition delay (2 s), and ${\mathbf{T}}_{\mathbf{P}}$ is the pre-defined time-to-peak delay. Bottom, right ventricle (RV) and pulmonary artery (PA) arterial input functions (AIF). ${\mathbf{t}}_{\mathbf{V}1}$ and ${\mathbf{t}}_{\mathbf{V}2}$ are the acquisition time of the first volume scan (V1) and the second volume scan (V2). The baseline volumes used to emulate the bolus-tracking are shown in blue circles.

In this study, we investigated the possibility of relating one-half the contrast bolus injection time (${{\varvec{T}}}_{{\varvec{I}}{\varvec{n}}{\varvec{j}}}/2$) and the time-to-peak delay (${{\varvec{T}}}_{{\varvec{P}}}$) of the AIF (Fig. 2). Such a relation was derived using the known contrast injection duration and the time-to-peak delay from the AIF, as described in Eq. 1. An empirically derived dispersion delay (${D}_{x}$) time constant was also introduced to describe the degree of the contrast bolus mixing. Such a factor is proportional to the physical distance between the contrast injection site and vessel of interest used for the AIF generation (Eq. 1)

1 $${T}_{P}= \alpha \times \frac{{ {\varvec{T}}}_{{\varvec{i}}{\varvec{n}}{\varvec{j}}}}{2}+{D}_{x}$$

where $\boldsymbol{\alpha }$ is the coefficient of the relation between one-half the injection time (${{\varvec{T}}}_{{\varvec{I}}{\varvec{n}}{\varvec{j}}}/2$) and the time-to-peak (${{\varvec{T}}}_{{\varvec{P}}}$), ${{\varvec{D}}}_{{\varvec{x}}}$ is the dispersion delay time constant.

### Data pre-processing

The images from each contrast-enhanced CT acquisition were first registered using a non-rigid algorithm^18^. Regions-of-interest (circular region with a diameter of 10 mm) were placed in the right ventricle, pulmonary artery, and descending aorta to generate arterial input functions (AIFs, Fig. 2). Next, a gamma-variate fitting (LSQCurveFit; Matlab 2013a, MathWorks) was performed on each dataset to generate smooth continuous AIF curves. Next, the 3D lung parenchyma was semi-automatically segmented using a standard commercial software (ViTAL Images, Lung CT, Pulmonary Analysis Workflow; Canon Medical Systems) and was used for the whole-lung FPA perfusion measurement. Further, 3D-segmentted binary masks of approximately 800–1400 mm^3^ were generated to measure regional perfusion. In summary, nine segments were assessed for each animal, including one segment for the left cranial lobe, left lingula lobe, right cranial lobe, right middle lobe, and accessory lobe; two segments for the left and right caudal lobes.

### Optimal retrospective protocol

Using the continuous AIF curve by the gamma variate fitting, the optimal acquisition timing for the baseline volume scan (V1) was defined as the peak of the second derivative, indicating the AIF curve starts to rise (Fig. 2). The optimal acquisition timing for the second volume scan (V2) was then defined as the true peak of the gamma variate fit. The time-to-peak delay between V1 and V2 was then computed and then averaged over multiple acquisitions in each animal. The average time-to-peak delay was related to one-half of the contrast injection time through regression analysis for both pulmonary artery and descending aorta.

### Aim 2—prospective protocol simulation

Bolus-tracking (SureStart, Aquilion One, Canon Medical Systems, Tustin, CA) was simulated for the prospective acquisition of the first volume scan (V1) at the base of the pulmonary artery AIF. It should be noted that there is a scanner transition dependent time delay (TD) between bolus-tracking and the acquisition of the first volume scan, which is less than 2 s for most scanners^17^. Hence, in order to acquire V1 close to the baseline enhancement of the pulmonary artery, bolus tracking was done in the right ventricle (RV) instead of the pulmonary artery to trigger earlier and partially compensate for the scanner transition delay (Fig. 2). Further, in order to define the baseline enhancement of the blood pool, a minimum of three pre-contrast images were used to emulate bolus-tracking. Multiple offset thresholds above the baseline, e.g. 40, 60, 80, 100, 120 and 140 HU, were compared to optimize the acquisition of V1. In addition, the second volume scan (V2) was automatically chosen using the predicted time-to-peak delay that was defined in Eq. 1. Hence, the prospective timing protocol simulation is summarized in Eqs. 2 and 3 as:

2 $${{t}_{V1}= t}_{trigger}+TD$$

3 $${t}_{V2}= {t}_{V1}+{T}_{P}$$

where ${{\varvec{t}}}_{{\varvec{V}}1}$ and ${{\varvec{t}}}_{{\varvec{V}}2}$ are the acquisition time of the V1 and V2, ${{\varvec{t}}}_{{\varvec{t}}{\varvec{r}}{\varvec{i}}{\varvec{g}}{\varvec{g}}{\varvec{e}}{\varvec{r}}}$ is the triggering time determined by bolus-tracking in RV, ${\varvec{T}}{\varvec{D}}$ is the transition delay between the trigger of bolus-tracking and the acquisition of V1, and ${{\varvec{T}}}_{{\varvec{P}}}$ is the predicted time-to-peak between the trigger and the peak of the AIF (Fig. 2). For clinical application, baseline (V1) and peak enhancement (V2) volume scans can be automatically acquired with predetermined delay times in a single protocol. The delay time for acquisition of V1 is a scanner dependent transition delay time (TD), which is less than 2 s for most scanners. The delay time for acquisition of V2 is calculated using contrast injection time.

### Two-volume FPA CT perfusion measurement

**Figure 3:**
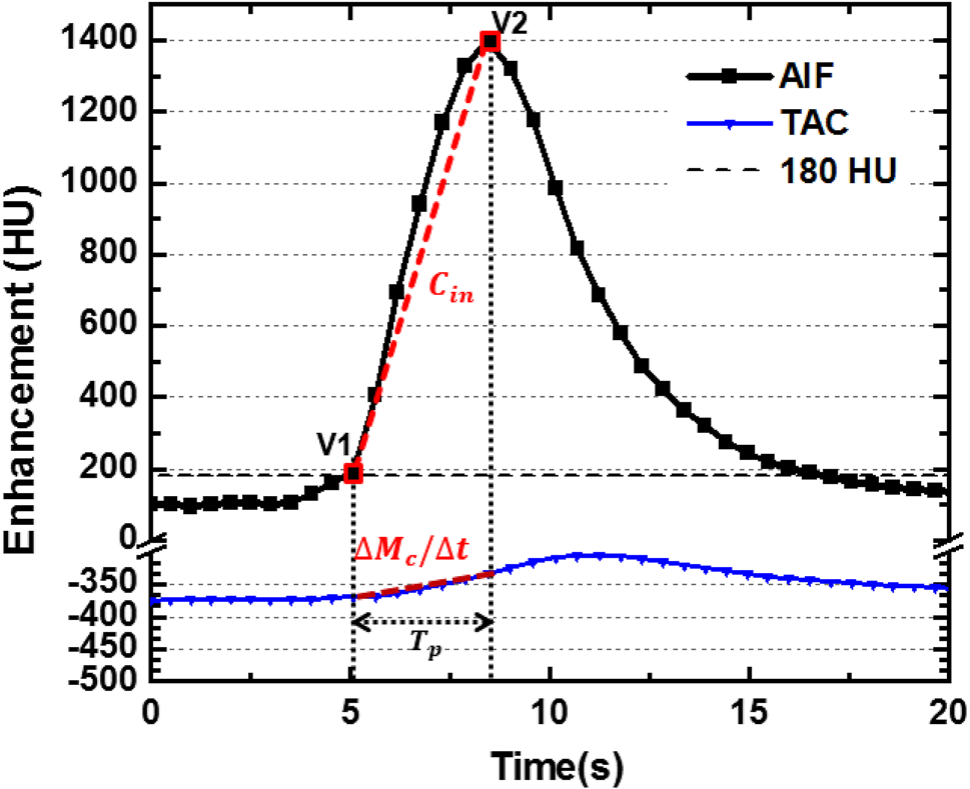
Two-volume FPA perfusion protocol. The integrated contrast enhancement change (${\Delta \mathrm{M}}_{\mathrm{c}}/\Delta \mathrm{t}$) within the lung compartment is measured by the tissue time attenuation curve (TAC, blue line). The average input concentration (${\mathrm{C}}_{\mathrm{in}}$) is estimated from the pulmonary arterial input function (AIF, black line) at V1 and V2. ${\mathrm{T}}_{\mathrm{p}}$ is the time-to-peak delay.

First-pass analysis has previously been used for blood flow measurement^19,20^. Assuming no contrast leakage over the measurement period (${[t}_{V1}, {t}_{V2}]$), the whole-lobe compartment is used to calculate the integrated contrast mass change in the perfusion bed (${\boldsymbol{\Delta }{\varvec{M}}}_{{\varvec{c}}}$**/**$\Delta {\varvec{t}}$) between V1 and V2^9,15^. The average input contrast concentration (${{\varvec{C}}}_{{\varvec{i}}{\varvec{n}}}$) of the pulmonary artery is also calculated between V1 and V2 (Fig. 3). Thus, the blood flow (${{\varvec{Q}}}_{{\varvec{a}}{\varvec{v}}{\varvec{e}}}$) is represented by^9,15^:

4 $${{\varvec{Q}}}_{{\varvec{a}}{\varvec{v}}{\varvec{e}}}=\frac{1}{{{\varvec{C}}}_{{\varvec{i}}{\varvec{n}}}}\boldsymbol{ }\frac{{\boldsymbol{\Delta }{\varvec{M}}}_{{\varvec{c}}}}{\Delta t}$$

where ${C}_{in}$ is the average input concentration, ${\boldsymbol{\Delta }{\varvec{M}}}_{{\varvec{c}}}$**/**$\Delta {\varvec{t}}$ is the rate of contrast mass change between ${t}_{V1}$ and ${t}_{V2}$, $\Delta t= {t}_{V2}-{t}_{V1}$. Finally, the regional perfusion of each 3D-segment is calculated and compared between the prospective and the reference retrospective two-volume pulmonary perfusion technique, where the retrospective two-volume perfusion technique was previously validated against fluorescent microspheres^15^.

### Cardiac output estimation

Since the pulmonary circulation carries the entire cardiac output (CO) from the right ventricle to the supply the lung, CO can be approximately estimated by the total pulmonary blood flow^21^ (Eq. 6).

Based on Eq. 4, the average contrast concentration change (${\boldsymbol{\Delta }{\varvec{M}}}_{{\varvec{c}}}$**)** within the entire compartment is proportional to the average pulmonary blood flow (${{\varvec{Q}}}_{{\varvec{a}}{\varvec{v}}{\varvec{e}}}$), the contrast concentration change per voxel (${\boldsymbol{\Delta }{\varvec{M}}}_{{\varvec{x}},{\varvec{y}},{\varvec{z}}}$) can be used to define pulmonary blood flow on a voxel-by-voxel basis (${{\varvec{Q}}}_{{\varvec{x}},{\varvec{y}},{\varvec{z}}}$) as:

5 $${{\varvec{Q}}}_{{\varvec{x}},{\varvec{y}},{\varvec{z}}}={{\varvec{Q}}}_{{\varvec{a}}{\varvec{v}}{\varvec{e}}}\frac{\Delta {{\varvec{M}}}_{{\varvec{x}},{\varvec{y}},{\varvec{z}}}}{{\boldsymbol{\Delta }{\varvec{M}}}_{{\varvec{c}}}}$$

Thus, the total pulmonary blood flow (${{\varvec{Q}}}_{{\varvec{p}}{\varvec{a}}}$), which is also the cardiac output (CO), is the summation of blood flow into the segmented voxels in the lung tissue:

6 $${{\varvec{C}}{\varvec{O}}\boldsymbol{ }\approx \boldsymbol{ }{\varvec{Q}}}_{{\varvec{p}}{\varvec{a}}}={\sum }_{k=0}^{n}{\boldsymbol{ }{\varvec{Q}}}_{{\varvec{x}},{\varvec{y}},{\varvec{z}}}$$

### Radiation dose

The CT dose index (${\mathrm{CTDI}}_{\mathrm{vol}}^{32}$, mGy) and the dose-length product (DLP, mGy∙cm) were recorded for each two-volume acquisition. Size-specific dose estimates (SSDE, mGy) were also calculated to account for the effective diameter of each swine^22^.

### Statistical approach

For the time-to-peak estimation, the empirical time-to-peak delays in the pulmonary artery and descending aorta were related to one-half the contrast injection time through linear regression analysis, where the root-mean-square-error (RMSE) and root-mean-square-deviation (RMSD) of the function were also calculated. The V2 acquisition time and contrast enhancement determined by the prospective protocol simulation were then compared with the actual peak time and the actual peak enhancement using paired sample t-testing (SPSS, version 22, IBM, Armonk, NY). Finally, simulated prospective two-volume perfusion measurements were quantitatively compared to the corresponding retrospective perfusion measurements through regression, Bland–Altman, RMSE, RMSD, and Lin’s concordance correlation coefficient (CCC).

## Results

### General data and radiation dose exposure

A total of 24 swine with an average weight of 48.5 ± 14.3 kg (25–91 kg) and an average heart rate of 89.5 ± 15.0 bpm were used for this study. In total, 141 successful injections were included for the time-to-peak prediction study (Fig. 1). Overall, the contrast injection durations ranged from 2 to 15 s and the cardiac outputs ranged from 1.4 to 5.1 L/min. The average ${\mathrm{CTDI}}_{\mathrm{vol}}^{32}$ and SSDE for each dynamic perfusion CT acquisition were 258.2 and 427.3 mGy, respectively. For prospective perfusion measurement using only two volumes, the average ${\mathrm{CTDI}}_{\mathrm{vol}}^{32}$ and SSDE were estimated to be 28.4 and 47.0 mGy, respectively.

### Aim 1—time-to-peak validation

**Figure 4:**
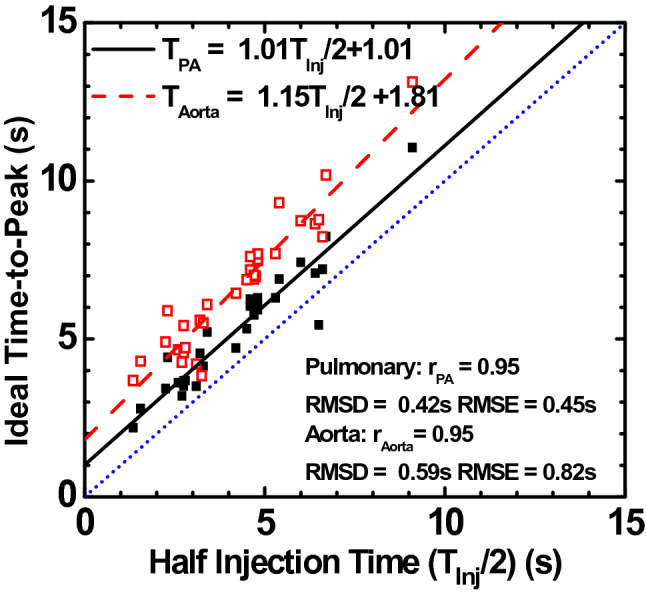
Time-to-peak delays in the pulmonary artery and descending aorta. Gamma-fit ideal time-to-peak delays were compared to the one-half injection time in all experimental animal data. Pulmonary arterial time-to-peak (black) and the aortic time-to-peak (red) are paralleled with different interceptions (dispersion factor). The blue dot line is the unity line. Gamma fit time-to-peak (*T*_*PA*_*, T*_*A*_) is defined as the time between the peak of the second derivative of the gamma fit and the true peak of the gamma fit, respectively. *T*_*Inj*_: contrast injection time; RMSE: root-mean-square-error; RMSD: root-mean-square-deviation; r: Pearson correlation coefficient.

The time-to-peak in the pulmonary artery (T_PA_) and descending aorta (T_A_) were related to one-half the contrast injection time by ${T}_{PA}=1.01\frac{{T}_{Inj}}{2}+1.01$(r = 0.95, RMSE = 0.45 s, RMSD = 0.42 s) and ${T}_{A}=1.15\frac{{T}_{Inj}}{2}+1.81$ (r = 0.95, RMSE = 0.82 s, RMSD = 0.59 s), respectively (Fig. 4). The intercepts correspond to organ-specific dispersion delay time constants (${D}_{x}$ in Eq. 1).

### Aim 2—prospective protocol simulation

A total of 60 CT acquisitions from 14 swine were used for the prospective perfusion measurements with bolus-tracking simulation. For each of the triggering offsets, the pulmonary artery enhancement and acquisition time of the simulated volume scans were compared to the optimal volume scans, as shown in Tables 2 and 3. To acquire V1 at a relatively low contrast enhancement, the triggering offset of 60HU in the RV was used in this prospective perfusion validation.

**Table 2:** Simulated prospective acquisition time versus optimal acquisition time.

Triggering offsets (HU)	V1			V2		
	Time difference (s)	RMSE	*P* value	Time difference (s)	RMSE	*P* value
40	0.61 ± 0.61	0.86	< 0.05	− 0.61 ± 0.98	1.16	< 0.05
60	0.87 ± 0.66	1.03	< 0.05	− 0.34 ± 0.96	1.02	< 0.05
80	1.05 ± 0.66	1.24	< 0.05	− 0.17 ± 0.90	0.91	0.252
100	1.20 ± 0.69	1.37	< 0.05	− 0.03 ± 0.85	0.85	0.921
120	1.38 ± 0.72	1.56	< 0.05	0.16 ± 0.83	0.84	0.056
140	1.53 ± 0.77	1.70	< 0.05	0.31 ± 0.82	0.88	< 0.05

The “Time Difference” shows the time difference between each bolus-tracking simulation and the optimal acquisition timing. The optimal acquisition timing is obtained from the gamma variate fitting AIF curve, where V1 at the second derivative peak of AIF and V2 at the AIF peak.

*RMSE* root mean square error. If the p-value is 0.05 or lower, the result is considered as significant difference.

**Table 3:** Simulated prospective enhancement versus optimal enhancement in the pulmonary artery.

Triggering offsets (HU)	C_V1_		C_V2_		C_V2_–C_V1_
	Enhancement (HU)	RMSE	Enhancement (HU)	RMSE	Difference (∆HU)
Optimal	228.8 ± 92.5	–	963.1 ± 294.4	–	735.1 ± 240.0
40	340.1 ± 151.9	161.0	923.1 ± 302.8	77.8	583.0 ± 295.5
60	389.1 ± 173.4	214.9	914.6 ± 311.2	96.4	525.6 ± 337.8
80	422. 5 ± 172.4	237.7	915.3 ± 316.2	97.8	493.6 ± 332.9
100	446.3 ± 183.2	255.9	914.9 ± 319.9	98.3	468.4 ± 334.5
120	486. 3 ± 187.4	300.0	900.0 ± 332.5	122.3	413.5 ± 357.5
140	514. 5 ± 190.5	325.3	891.4 ± 330.5	134.6	377.3 ± 364.0

The “Optimal Enhancement” in the pulmonary artery is the simulated result from the gamma variate fitting curve. Specifically, C_V1_ was calculated as the average enhancement within the PA region at the time of the second derivative peak, C_V2_ was the average enhancement within the PA region at the time of peak enhancement. The root-mean-square-error (RMSE) between each triggering protocol and the ideal enhancement was also calculated.

The change in contrast enhancements (**∆HU**) between C_V1_ and C_V2_ is shown in the last column. *P* values in all comparisons are less than 0.001. Blood flow is measured based on **∆HU.** Therefore, it is best for C_V1_ to be as low as possible and C_V2_ to be as high as possible.

### Two-volume FPA CT perfusion measurement

**Figure 5:**
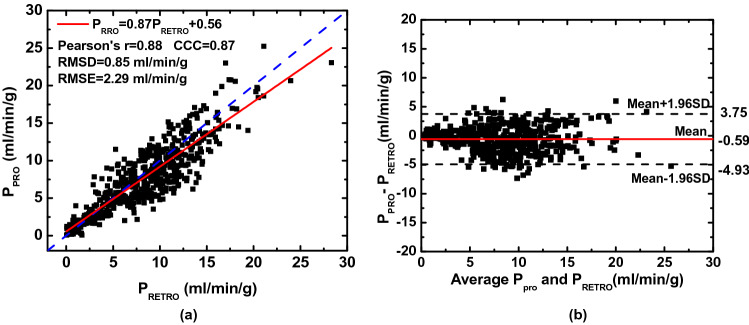
(**a**) Regression analysis comparing the result of simulated two-volume prospective perfusion measurements (${\mathrm{P}}_{\mathrm{PRO}}$) to the corresponding reference retrospective perfusion measurements ${(\mathrm{P}}_{\mathrm{RETRO}}$). Each data point represents a 3D perfusion segment from the swine. For the retrospective assessment, the optimal V1 and V2 were selected at the base and peak of the AIF from the gamma fitting curve. For the prospective measurement, bolus-tracking simulation was conducted in the right ventricle within triggering threshold at 60 HU above the blood pool enhancement. (**b**) Bland–Altman analysis was performed with the limits of agreement. *CCC:* concordance correlation coefficient, *RMSD:* root-mean-square deviation, *RMSE:* root-mean-square error, *SD:* standard deviation.

**Figure 6:**
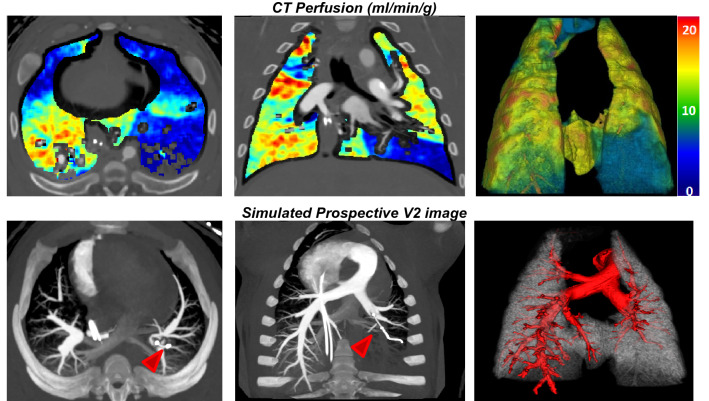
Representative CT perfusion maps and the prospectively predicted V2 image. Top row: Axial, coronal, and 3D posterior views in the presence of balloon occlusions are shown. The color bar on the right indicates the perfusion in the range from 0 to 20 ml/min/g. Bottom row: Axial, oblique-coronal CT angiography using maximum intensity projection reconstruction with 20 mm thickness and 3D pulmonary arteries extraction (Using V2). Red arrows point at the angioplasty balloon.

The perfusion assessments were based on a 9-segment model with a total of 540 lung segments. The mean perfusion of the retrospective and the simulated prospective measurements were 8.43 $\pm$ 4.54 ml/min/g and 7.84 $\pm$ 4.47 ml/min/g (*P* < 0.001), respectively. The simulated prospective FPA perfusion (P_PRO_) were related to reference retrospective perfusion (P_RETRO_) measurements by P_PRO_ = 0.87P_RETRO_ + 0.56 (Pearson’s r = 0.88, RMSD = 0.85 ml/min/g, RMSE = 2.29 ml/min/g), with a concordance correlation coefficient of 0.87 (Fig. 5a). The corresponding Bland–Altman analyses is also displayed in Fig. 5b. The linear regression results of perfusion measurements for individual lobes are shown in Table 4. There is no evident bias between lobes except for a larger error in the accessory lobe caused by the highly attenuating iodine in the vena cava. Representative examples of prospective two-volume FPA perfusion maps and the V2 image for one acquisition are shown in Fig. 6. Both the pulmonary arterial occlusion and the perfusion defect in the distal left caudal lobe can be visualized by a single contrast injection.

**Table 4:** Regression of simulated two-volume prospective FPA perfusion versus retrospective FPA perfusion.

Segments (n)	Slope	Intercept	Pearson r	CCC	RMSE (ml/min/g)	RMSD (ml/min/g)	*P* value
All (540)	0.87 (0.83, 0.90)	0.56 (0.18, 0.93)	0.88 (0.85, 0.89)	0.87 (0.85, 0.89)	2.29	0.85	< 0.001
R_Cranial (60)	0.82 (0.66, 0.98)	1.13 (− 0.42, 2.68)	0.81 (0.77, 0.83)	0.80 (0.77, 0.83)	2.23	0.83	0.051
R_Middle (60)	0.77 (0.62, 0.93)	0.91 (− 0.26, 2.07)	0.80 (0.77, 0.83)	0.78 (0.75, 0.81)	2.19	0.90	0.019
R_Caudal (120)	0.91 (0.91, 1.00)	0.49 (− 0.69, 1.66)	0.86 (0.83, 0.88)	0.85 (0.82, 0.87)	2.56	0.68	0.022
AL (60)	0.69 (0.53, 0.84)	1.90 (0.65, 3.16)	0.76 (0.72, 0.79)	0.74 (0.71, 0.78)	2.20	1.09	0.150
L_Cranial (60)	0.84 (0.69, 0.99)	0.87 (− 0.69, 2.42)	0.82 (0.81, 0.86)	0.81 (0.78, 0.84)	1.87	0.85	0.036
L_Lingula (60)	0.80 (0.66, 0.93)	0.63 (− 0.37, 1.64)	0.82 (0.79, 0.85)	0.81 (0.78, 0.84)	1.87	1.00	0.001
L_Caudal (120)	0.84 (0.77, 0.91)	0.50 (− 0.10, 1.08)	0.92 (0.90, 0.93)	0.91 (0.89, 0.92)	2.31	1.08	0.003

Data in parentheses are 95% confidence intervals. *P* values less than 0.05 indicate significant difference.

*CCC:* concordance correlation coefficient, *RMSD:* root-mean-square deviation, *RMSE:* root-mean-square error, *AL:* accessory lobe, *R:* right lung, *L:* left lung.

## Discussion

In this study, the time-to-peak delay of the pulmonary and aortic AIFs were evaluated in animals with a range of body weights (25–91 kg), contrast doses (20–100 mL), injection durations (2–15 s), and cardiac outputs (1.4–5.1 L/min). The results indicate that the injection duration is the most significant injection-related parameter impacting the bolus time-to-peak, particularly in the case of short contrast injection duration. Furthermore, the regional perfusion results show good correlation between the simulated prospective FPA measurements and the optimal retrospective FPA measurements. Such findings indicate that the proposed prospective timing protocol can potentially be used for accurate, prospective, two-volume FPA perfusion measurement.

Existing dynamic CT perfusion techniques, such as the maximum slope model and the deconvolution model, require the entire contrast pass curve for perfusion measurement resulting in a high effective radiation dose^3,7,23–27^. Previous reports have shown that the reduction of temporal sampling frequency reduces the accuracies of these current techniques^28^. Although previous reports have shown that the FPA technique can accurately measure the perfusion using only two volume scans as validated using microspheres^9,15^, the prospective acquisition of the two volume scans is challenging. With the knowledge of contrast timing information in advance, the prospective implementation of the two-volume perfusion technique can accurately measure the pulmonary perfusion while substantially reducing the radiation dose. By reducing the total number of volume scans from twenty to two, such prospective perfusion technique also can significantly reduce breath-hold time for patients with pulmonary disease, preventing the potential respiratory motion artifacts.

Bolus tracking is commonly used to detect the contrast arrival time within a region of interest in an input artery. A fixed time delay is currently used to estimate the time to peak of the contrast bolus. In this study, we used a injection-specific time-to-peak estimation. Prior to recirculation phase of the contrast agent bolus passage, the geometry of the arterial input function (AIF) is predominantly determined by the contrast bolus injection geometry and the bolus dispersion within the circulatory system, given a short contrast injection duration (< 15 s)^17,29^. Specifically, the initial approximate rectangular geometry of an undiluted contrast bolus injection will dilute and disperse into a contrast pass curve, where the area under the curve remains conserved and the width of the curve remains proportional to the amount of the contrast volume injected at a fixed rate^30,31^. Hence, despite contrast mixing and hemodynamic perturbation, the dispersion of the bolus primarily occurs at its temporal edges or tails; hence, the center of the AIF has the maximal contrast attenuation. As such, the time-to-peak delay from the AIF was pre-determined using one-half of contrast injection duration.

Our results demonstrate that the injection duration can be used to predict the time-to-peak for different injection rates and volumes, and are therefore in agreement with a previous report indicating that the scanning delay for the aortic peak is primarily affected by the injection duration^17^. To generalize our timing prediction theory to clinical patients with different cardiac outputs, different levels of pulmonary arterial occlusions were generated in our swine model, resulting in a substantial decrease of cardiac outputs. Fortunately, the time-to-peak delays under different scenarios were all closely related to one-half of the injection duration (Pearson’s r = 0.95), indicating the robustness of the timing theory. Such results may also have important implications for optimal CT pulmonary angiography (CTPA), as the optimal time-to-peak delay can be predicted using the contrast injection time interval. Although further validation remains necessary, the proposed time-to-peak prediction may result in an improved contrast opacification in CTPA and visualization of the vasculature.

Finally, the proposed two-volume perfusion quantifies absolute perfusion (in ml/min/g). Quantitative absolute perfusion has the potential for improved assessment of the degree of perfusion defect. Hence, the dynamic two-volume perfusion technique can potentially be an alternative to the standard dynamic perfusion CT by providing functional assessment of pulmonary diseases, such as pulmonary embolism and chronic thromboembolic pulmonary hypertension, at a reduced radiation dose. To further optimize the radiation dose, the V1 can be acquired using a low-dose volume scan, while V2 can be acquired using standard dose for CT pulmonary angiography (CTPA). Consequently, the combination of V1 and V2 can be used for perfusion measurement, while V2 itself can also be used as the CTPA. Hence, both morphological and functional assessment can be provided via a single contrast injection at a radiation dose that is only slightly higher than the standard CT angiography dose.

This study has several limitations. First, most of the swine used in the study were relatively small as compared to the average size of a patient. Additional studies may be necessary for larger patient sizes (> 90 kg) to further validate the dispersion delay time constant robustness. Second, retrospective FPA perfusion measurement was used for validation of the simulated prospective two-volume perfusion measurement. However, the accuracy of the retrospective FPA perfusion technique has previously been validated using fluorescent microspheres as the reference standard^15^. Third, although the time-to-peak prediction has not been validated in patients with various cardiopulmonary conditions (such as acute pulmonary embolism, pulmonary hypertension, and heart failure), the time-to-peak prediction was tested following different levels of occlusion in the pulmonary artery of a swine model. Additionally, such timing protocol remained robust over a wide range of cardiac outputs. However, future studies will need to test this technique in the presence of chronic embolism and collateral flow. Fourth, since the scanner transition delay time is manufacturer-specific, the bolus tracking trigger location and threshold have not been optimized for other CT scanners. A long scanner transition time delay after triggering may result in a late acquisition of V1. This could potentially be addressed by performing bolus tracking in the right ventricle rather than pulmonary artery. Alternatively, the contrast arrival time can be determined using a diluted test bolus acquisition^32^, although the contrast and radiation dose will be slightly increased. The simulated pharmacokinetic global circulation models can also be helpful in prediction of contrast timing^33^. Finally, the optimal prospective timing protocol was developed and assessed empirically; hence, the diagnostic performance of the two-volume FPA pulmonary perfusion technique with simultaneous CTPA (using the V2 volume scan), will require further studies.

In conclusion, an optimal timing protocol for a low-dose, two-volume dynamic CT pulmonary perfusion technique was developed and validated in a swine model. By using the dynamic bolus-tracking and time-to-peak delay estimation, the optimal timing protocol resulting in robust acquisition of the first volume scan at the base of the AIF and the second volume scan at the peak of AIF. Such finding enables a practical, low-dose, two-volume dynamic CT perfusion technique that may potentially act as a perfusion-based biomarker for stratifying the severity, prognosis, and follow-up in patients with pulmonary embolism and other pulmonary pathologies.
